# An Exploratory Study Provides Insights into MMP9 and Aβ Levels in the Vitreous and Blood across Different Ages and in a Subset of AMD Patients

**DOI:** 10.3390/ijms232314603

**Published:** 2022-11-23

**Authors:** Savannah A. Lynn, Flavie Soubigou, Jennifer M. Dewing, Amanda Smith, Joanna Ballingall, Thea Sass, Isabela Nica, Catrin Watkins, Bhaskar Gupta, Hussein Almuhtaseb, Stephen C. Lash, Ho Ming Yuen, Angela Cree, Tracey A. Newman, Andrew J. Lotery, J. Arjuna Ratnayaka

**Affiliations:** 1 Clinical and Experimental Sciences, Faculty of Medicine, University of Southampton, MP806, Tremona Road, Southampton SO16 6YD, UK; 2 Eye Unit, University Hospital Southampton NHS Foundation Trust, Southampton SO16 6YD, UK; 3 Primary Care, Population Sciences and Medical Education, Faculty of Medicine, University of Southampton, MP 801, Tremona Road, Southampton SO16 6YD, UK

**Keywords:** MMP9, amyloid beta (Aβ), age-related macular degeneration (AMD), biomarkers, lifecourse, lifestyle, smoking, mean arterial pressure

## Abstract

Matrix metalloproteinase-9 (MMP9) and total amyloid-beta (Aβ) are prospective biomarkers of ocular ageing and retinopathy. These were quantified by ELISA in the vitreous and blood from controls (*n* = 55) and in a subset of age-related macular degeneration (AMD) patients (*n* = 12) for insights and possible additional links between the ocular and systemic compartments. Vitreous MMP9 levels in control and AMD groups were 932.5 ± 240.9 pg/mL and 813.7 ± 157.6 pg/mL, whilst serum levels were 2228 ± 193 pg/mL and 2386.8 ± 449.4 pg/mL, respectively. Vitreous Aβ in control and AMD groups were 1173.5 ± 117.1 pg/mL and 1275.6 ± 332.9 pg/mL, whilst plasma Aβ were 574.3 ± 104.8 pg/mL and 542.2 ± 139.9 pg/mL, respectively. MMP9 and Aβ showed variable levels across the lifecourse, indicating no correlation to each other or with age nor AMD status, though the smaller AMD cohort was a limiting factor. Aβ and MMP9 levels in the vitreous and blood were unrelated to mean arterial pressure. Smoking, another modifiable risk, showed no association with vitreous Aβ. However, smoking may be linked with vitreous (*p* = 0.004) and serum (*p* = 0.005) MMP9 levels in control and AMD groups, though this did not reach our elevated (*p* = 0.001) significance. A bioinformatics analysis revealed promising MMP9 and APP/Aβ partners for further scrutiny, many of which are already linked with retinopathy.

## 1. Introduction

The use of different types of biomarkers or combinations thereof presents an opportunity to evaluate ocular health across the lifespan and to predict the odds of developing blinding conditions or even gain insights into their rate of progression. The better the specificity of the biomarkers, the more accurately they can be used in combination with other indicators to determine the odds of developing complex retinopathies such as age-related macular degeneration (AMD). Signs of ageing and retinopathy can be ascertained by non-invasive retinal assessments such as spectral domain optical coherence tomography (SD-OCT) and fluorescence angiography, which provide structural readouts of individual retinal layers including the integrity of the retinal vasculature. Techniques such as fundus autofluorescence offer insights into the biochemistry of the retinal pigment epithelium (RPE), whilst microperimetry allows correlative studies between macular pathology and functional abnormalities. Collectively, these non-invasive methods are used to classify AMD [1,2].

The measurement of target molecules directly from the patient’s biological material, however, provides the opportunity to refine these assessments even further. Hence, there is considerable interest in correlating specific proteins from ocular fluids such as the vitreous and aqueous humour, as well as from blood, with ageing and with distinct stages of AMD. These efforts have been facilitated by advances in the sensitivities of enzyme-linked immunosorbent assays (ELISAs), multiplex methodologies and other tools, which can yield valuable information from increasingly smaller sample volumes with high levels of reproducibility. Nonetheless, efforts to correlate disease-linked proteins across the lifecourse or with specific stages of AMD have not proved straightforward, as genetic backgrounds and lifestyle risk factors such as smoking and types of diet as well as other co-morbidities present a mixture of confounding variables that must be taken into consideration.

In this exploratory study, we set out to gain further insights into the constraints of evaluating these parameters by quantifying the immature and active levels of matrix metalloproteinase 9 (MMP9), an AMD risk factor, in the vitreous and blood across the lifecourse and in a subset of AMD patients. We also quantified total levels of the Alzheimer’s-linked amyloid beta (Aβ) proteins in the vitreous and plasma in both groups, which is of considerable interest given its association with ageing and retinopathy [3,4], as well as due to recent discoveries demonstrating close links between retinal and neuropathologies such as Alzheimer’s disease (AD) and dementia [5,6,7,8,9]. Our findings from a cohort of 55 control subjects across different ages and in a subset of 12 AMD patients revealed that MMP9 and Aβ levels in the ocular compartment and in the systemic circulation do not correlate with age or with AMD. There was also no correlation in MMP9 and Aβ between the retinal environment (vitreous) and systemic circulation (serum and plasma). Furthermore, no obvious association between MMP9 and Aβ levels in the ocular or systemic circulation could be attributed to mean arterial pressure in either control or AMD groups. Smoking, another modifiable AMD risk factor, also showed no correlation with vitreous or plasma Aβ in either group. The extent of smoking, however, may have some relationship with vitreous or serum MMP9 levels in control and AMD groups. Collectively, our findings defined important inclusion and exclusion criteria for setting-up larger studies of this kind, and identified lifestyle and co-morbidity factors that should be taken into consideration, including the identity of other MMP9- and Aβ-interacting proteins, which could increase the odds of delineating key biomarkers of ageing vs. the risks of developing retinopathy.

## 2. Results

### 2.1. Scrutiny of the Control and AMD Cohorts Revealed a Mixed Picture of Age, Gender, Ocular History and Medication as Well as Lifestyle Demographics

This study was undertaken over a 5-year period and recruited 55 control subjects from vitreoretinal clinics (see Table 1 for inclusion criteria). These were patients that were scheduled for vitrectomy for an ocular diagnosis unrelated to AMD that does not influence ocular pathology. In addition, we included 12 patients with confirmed AMD who also attended these clinics. AMD patients were difficult to recruit, as their treatment does not require the specific removal of vitreous samples.

**Figure 1:**
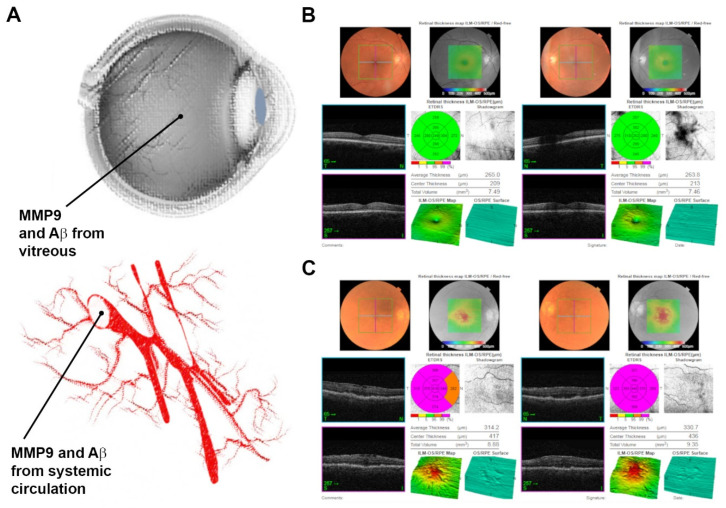
Experimental plan and representative retinal scans of study participants. (**A**) Schematic diagram of the privileged ocular compartment vs. the systemic circulation from which vitreous and blood samples were collected, respectively. Enzyme-linked immunosorbent assays (ELISAs) quantified total Aβ and immature/active MMP9 levels in the vitreous and blood (serum/plasma) samples. (**B**) Representative retinal scans of a participant in the control cohort showing funduscopy and optical coherence tomography (OCT) images. (**C**) Scans from a participant with geographic atrophy AMD. OCT scans show diffuse drusen at the level of the retinal pigment epithelium in each eye. Both eyes also show the presence of an epiretinal membrane.

Our work sought to evaluate whether levels of MMP9, a biomarker of AMD, or Aβ, which is an indicator of ageing as well as neurodegeneration in the retina and the brain, change with age and AMD status. We also assessed any associations these may have with lifestyle risk factors. Furthermore, we sought to gain insights into potential links between ocular biomarkers of ageing and disease vs. their relative concentrations in the systemic circulation (Figure 1A). Funduscopy and OCT images were obtained during appointments. Representative retinal scans of a control subject (Figure 1B) and an AMD patient (Figure 1C) are shown.

**Figure 2:**
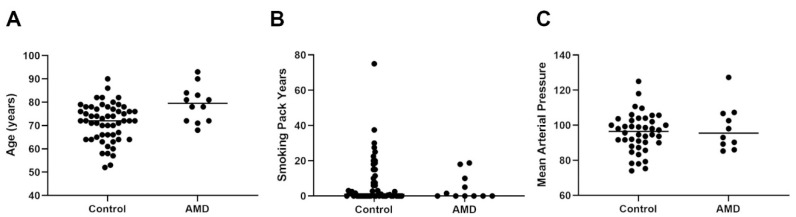
Characteristic features of the control and AMD cohorts. (**A**) The age distribution of control subjects (*n* = 55) attending vitreoretinal clinics alongside a subset of AMD patients (*n* = 12) were broadly similar, though ages in the latter group were clustered at the higher end of the spectrum. (**B**) A comparison of smoking pack years in the control (*n* = 29) and AMD (*n* = 6) cohorts showed no evidence of increased smoking in the latter group. A value of zero was recorded for participants who did not smoke, but provided this information. (**C**) A comparison of mean arterial pressure in the control (*n* = 42) and AMD (*n* = 10) groups indicated no differences.

Further information on the participants was obtained from patient records including age, gender, smoking status and smoking pack years, which indicates the amount a subject has smoked over a period of time. We also obtained a recording of mean arterial pressure, the primary cause of surgery, previous ocular history and medical history as well as medication (Supplementary Table S1). AMD patients consisted of those presenting with both dry/geographic atrophy (GA) and wet/neovascular (NV) forms of the disease. Medical records described the following phenotypes in our AMD cohort: early AMD (*n* = 4), GA (*n* = 7) and NV (*n* = 1). The age distribution amongst control subjects ranged from 52 to 90 years (mean: 71.3 years ± SEM 1.1; median: 72 years), whilst the age range in the AMD cohort was 68 to 93 years (mean: 79.25 years ± SEM 2.2; median: 79.5 years) (Figure 2A). The gender distribution amongst the control subjects was 38 females and 17 males, whilst the AMD cohort contained 7 females and 5 males. Scrutiny of smoking status revealed a mixed picture amongst control subjects, which was grouped as not declared (*n* = 1), never smoked (*n* = 23), ex-light smoker (*n* = 15), ex-heavy smoker (*n* = 8), light smoker (*n* = 2) and heavy smoker (*n* = 6). The smoking status in AMD patients was; never smoked (*n* = 6), ex-light smoker (*n* = 3) and ex-heavy smoker (*n* = 3) (Supplementary Table S1). Smoking pack years were recorded in 29 control subjects (mean: 7.7 ± 2.5 SEM; median: 1) and in 6 AMD patients (mean: 4.8 ± 3 SEM; median: 0.01) (Figure 2B). The mean arterial pressure was noted in 42 control subjects and ranged from 75.22–110.67 mm/Hg (mean: 95.7 mm/Hg ± 1.7 SEM; median: 96.5 mm/Hg). The mean arterial pressure was obtained from 10 AMD patients, which ranged between 85.33 and 127.33 mm/Hg (mean: 98.6 mm/Hg ± 4.1 SEM; median: 95.5 mm/Hg) (Figure 2C).

The primary causes of surgery amongst control subjects were as follows: cataract (CAT) (*n* = 10), epiretinal membranes (ERM) (*n* = 31), macular hole (MH) (*n* = 13), vitreous floaters (VF) (*n* = 3) and vitreomacular traction (VMT) (*n* = 7). Among the AMD cohort, this was recorded as CAT (*n* = 3), ERM (*n* = 7), MH (*n* = 2), retinal detachment (RD) (*n* = 1) and VMT (*n* = 2). Some individuals reported multiple conditions in both control and AMD groups (Supplementary Table S1). Scrutiny of previous ocular history among all participants revealed that some individuals had a history of these conditions but also pathologies such as glaucoma (GLA) and posterior vitreous detachment (PVD). Previous medical history and any medications were also recorded for all participants (Supplementary Table S1).

### 2.2. Aβ and MMP9 Levels in the Vitreous and Blood of Control Subjects and AMD Patients Showed Variable Levels across the Lifespan, Which Was Unrelated with Advanced Age or Retinopathy

**Figure 3:**
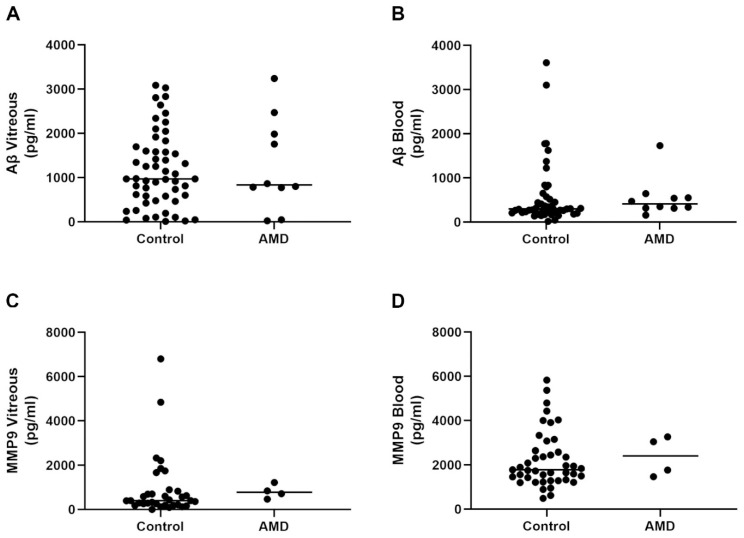
ELISA measurements of Aβ and MMP9 protein levels in the vitreous and blood. (**A**) Total Aβ levels in the vitreous of control subjects (*n* = 53) and AMD patients (*n* = 10), as well as (**B**) in the blood/plasma of control (*n* = 48) and AMD (*n* = 10) groups, showed a wide and overlapping distribution. (**C**) Immature and active MMP9 levels in the vitreous of control (*n* = 34) and AMD patients (*n* = 4), as well as (**D**) in the blood/serum of control subjects (*n* = 43) and AMD patients (*n* = 4), showed a similar pattern of distribution.

After obtaining vitreous and blood samples from participants, we quantified the amount of total Aβ (Aβ_1–x_) by ELISA. The quantity of Aβ in the vitreous of the control subjects ranged from 12.0 to 3086.91 pg/mL (mean: 1173.5 pg/mL ± 117.1 SEM; median: 971.5 pg/mL). Vitreous Aβ levels in AMD patients also displayed a wide distribution, ranging between 26.08 and 3240.12 pg/mL (mean: 1275.6 pg/mL ± 332.9 SEM; median: 833.4) (Figure 3A and Supplementary Table S1). The distribution of Aβ levels in the blood of the control subjects varied from 44.18 to 3102.18 pg/mL (mean: 574.3 pg/mL ± 104.8 SEM; median: 301.1). Aβ levels in the blood of the AMD patients ranged from 156.9 to 1732.15 pg/mL (mean: 542.2 pg/mL ± 139.9 SEM; median: 411.9) (Figure 3B and Supplementary Table S1). Measurements were obtained from a majority of subjects and only omitted when Aβ concentrations were below the threshold of the ELISA standard curve. Next, we used an ELISA to quantify the amount of MMP9 in the vitreous of the subjects in the control cohort. MMP9 concentrations showed a wide distribution, which ranged between 0.45 and 4844.8 pg/mL (mean: 932.5 pg/mL ± 240.9 SEM; median: 400.7). The distribution of MMP9 in the vitreous of AMD patients varied from 469 to 1224.9 pg/mL (mean: 813.7 pg/mL ± 157.6 SEM; median: 780.5) (Figure 3C and Supplementary Table S1). We also quantified levels of MMP9 in the blood of the control subjects, which ranged between 487.8 and 5831.7 pg/mL (mean: 2228 pg/mL ± 193 SEM; median: 1780.7). MMP9 levels in the blood of the AMD patients were between 1468.9 and 3264.2 pg/mL (mean: 2386.8 pg/mL ± 449.4 SEM; median: 2407) (Figure 3D and Supplementary Table S1). MMP9 levels were thus recorded from a majority of the participants and only omitted when concentrations were below the threshold of the ELISA standard curve.

**Figure 4:**
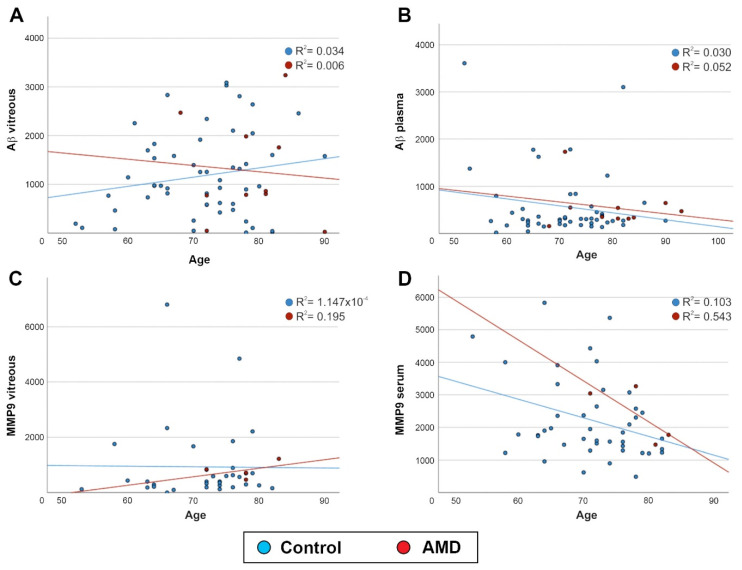
Correlation analysis of MMP9 and Aβ levels in the vitreous and blood across mid-life to old age. (**A**) A comparison of total Aβ concentrations in the vitreous (control: *n* = 54, AMD: *n* = 10; *p* = 0.963) or (**B**) plasma (control: *n* = 48, AMD: *n* = 10; *p* = 0.694) with age revealed no associations in controls or in a subset of AMD patients. Similarly, a comparison of MMP9 levels in (**C**) the vitreous (control: *n* = 35, AMD: *n* = 4; *p* = 0.882) or (**D**) serum (control: *n* = 43, AMD: *n* = 4; *p* = 0.37) with age showed no associations in either group.

Correlation analyses, which jointly account for AMD and control patients in the statistical model, revealed no significant association between age and Aβ levels in the vitreous (*p* = 0.963) or blood (*p* = 0.694) from the controls or AMD patients (Figure 4A,B). Similarly, age showed no correlation with the MMP9 levels in the vitreous (*p* = 0.882) or blood (*p* = 0.37) in either group (Figure 4C,D).

**Figure 5:**
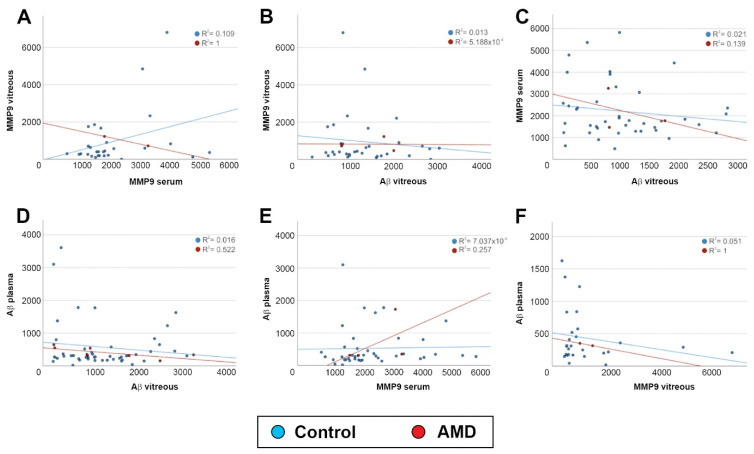
Correlation analysis of MMP9 vs. Aβ. (**A**) MMP9 levels in the vitreous vs. MMP levels in serum were compared to determine any association of this AMD risk factor between the ocular compartment vs. the systemic circulation. No association was found in either control subjects or in AMD patients (control: *n* = 29, AMD: *n* = 2; *p* = 0.09). (**B**) A comparison of MMP9 levels in the vitreous vs. Aβ vitreous levels also showed no association in control subject or in AMD patients (control: *n* = 34, AMD: *n* = 4; *p* = 0.53). (**C**) Similarly, there was no association between serum MMP9 levels with Aβ vitreous levels in controls or in AMD patients (control: *n* = 41, AMD: *n* = 3; *p* = 0.33). (**D**) Aβ levels in the plasma vs. Aβ levels in the vitreous were compared to determine any association in this risk factor between the ocular compartment vs. the systemic circulation. No association was found in either control subjects or in AMD patients (control: *n* = 46, AMD: *n* = 8; *p* = 0.32). (**E**) We also found no correlation between Aβ levels in plasma with MMP9 levels in serum of controls or AMD patients (control: *n* = 41, AMD: *n* = 4; *p* = 0.73). (**F**) Similarly, no correlation was observed between Aβ levels in plasma with MMP9 levels in the vitreous of control subjects or AMD patients (control: *n* = 27, AMD: *n* = 2; *p* = 0.25).

Next, we tested if Aβ vs. MMP9 levels in the ocular compartment and systemic circulation bore any correlation to each other. No association was found between MMP9 levels of the vitreous vs. serum (*p* = 0.09) for the control subjects or AMD patients (Figure 5A), or between vitreous MMP9 levels vs. vitreous Aβ levels (*p* = 0.53) for either group (Figure 5B). There was also no correlation between the serum MMP9 levels vs. Aβ levels in the vitreous for the controls (*p* = 0.33) or AMD patients (Figure 5C). The plasma Aβ levels showed no correlation with the vitreous Aβ concentrations (*p* = 0.32) for either group (Figure 5D). Comparison of plasma Aβ levels vs. serum MMP9 levels showed no correlation (*p* = 0.73) for the controls or AMD patients (Figure 5E), nor were there any apparent relationships between the plasma Aβ levels vs. vitreous MMP9 concentrations (*p* = 0.25) for either group (Figure 5F).

### 2.3. Aβ and MMP9 Levels in the Vitreous and Blood Were Unrelated to Mean Arterial Pressure and Smoking

**Figure 6:**
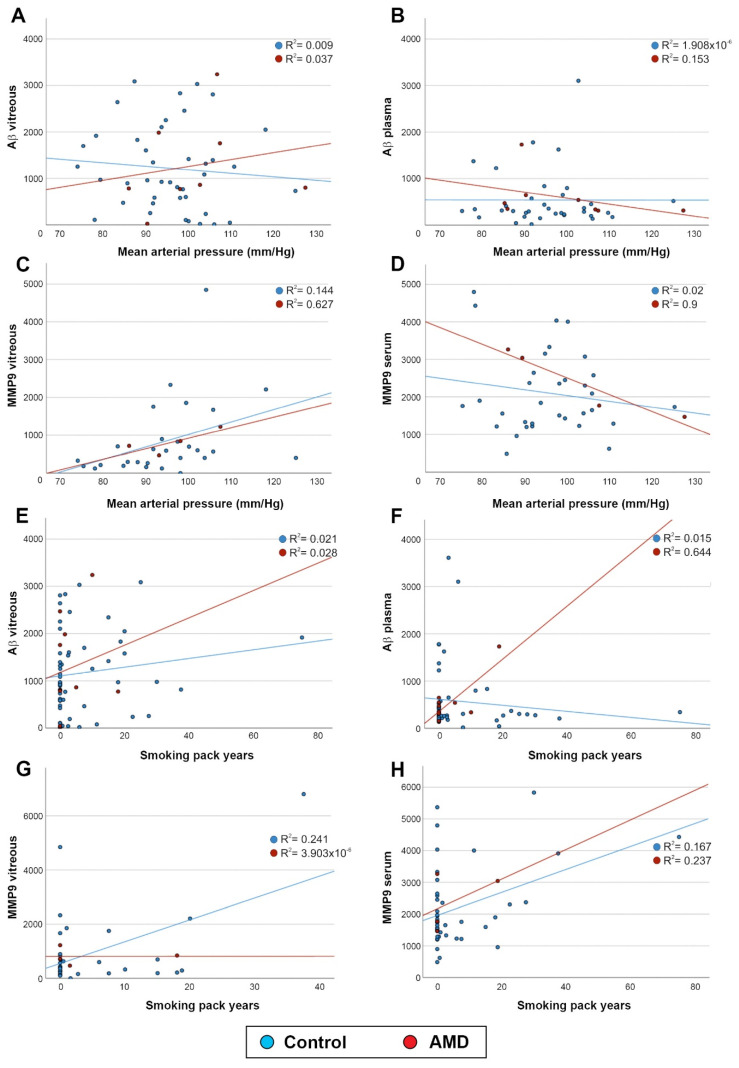
Correlation analysis of MMP9 and Aβ levels with mean arterial pressure and with the extent of smoking. (**A**) Evaluation of Aβ levels in the vitreous with mean arterial pressure showed no association in control subjects or in AMD patients (control: *n* = 42, AMD: *n* = 8; *p* = 0.791). (**B**) Similarly, Aβ levels in the systemic circulation had no correlation with mean arterial pressure in either group (control: *n* = 35, AMD: *n* = 8; *p* = 0.660). (**C**) A comparison of MMP9 levels in the ocular compartment with mean arterial pressure in control subjects and in AMD patients (control: *n* = 29, AMD: *n* = 4; *p* = 0.032) showed no correlation. (**D**) Similarly, MMP9 levels in the serum had no correlation with mean arterial pressure in either group (control: *n* = 33, AMD: *n* = 4; *p* = 0.152). We also studied the relationship between these biomarkers and the extent of smoking (smoking pack years). (**E**) Correlation analysis of vitreous Aβ levels with smoking pack years showed no association in control subjects or in AMD patients (control: *n* = 54, AMD: *n* = 10; *p* = 0.276). (**F**) We also found no correlation between systemic Aβ levels and smoking pack years in controls or in AMD patients (control: *n* = 47, AMD: *n* = 10; *p* = 0.582). (**G**) Evaluation of MMP9 levels in the vitreous with the extent of smoking indicated a possible association with both groups (control: *n* = 34, AMD: *n* = 4; *p* = 0.004), but one that did not reach a statistical threshold of *p* = 0.001. (**H**) Similarly, a possible link was observed between systemic MMP9 levels with smoking pack years in control and AMD patients (control: *n* = 42, AMD: *n* = 4; *p* = 0.005). However, this also failed to achieve a *p* = 0.001 threshold, inviting further scrutiny into this putative relationship.

Given that mean arterial pressure and smoking pack years provide important information on lifestyle risks, we evaluated if these demonstrated any correlation with MMP9 and Aβ levels in the ocular compartment or in the systemic circulation. A comparison of vitreous Aβ levels vs. mean arterial pressure showed no correlation (*p* = 0.791) for control subjects or AMD patients (Figure 6A). Plasma Aβ levels also showed no correlation with mean arterial pressure (*p* =0.660) in either group (Figure 6B). We also found no discernible relationship between MMP9 levels in the vitreous and mean arterial pressure (*p* = 0.032) for controls or AMD patients (Figure 6C), or between serum MMP9 levels and mean arterial pressure (*p* = 0.152) in either group (Figure 6D). Next, we examined the relationship between vitreous Aβ levels and smoking pack years, which showed no correlation (*p* = 0.276) in control subjects or in AMD patients (Figure 6E). A comparison of plasma Aβ levels vs. smoking pack years also indicated no association (*p* = 0.582) in either group (Figure 6F). Similarly, vitreous MMP9 concentrations did not show a significant correlation with smoking pack years (*p* = 0.004) in controls and in AMD patients (Figure 6G). Serum MMP9 levels were also unrelated with smoking pack years (*p* = 0.005) in both groups (Figure 6H). For scrutinising all aforementioned comparisons, we increased the threshold of significance from *p* = 0.05 (Bonferroni) to *p* = 0.001, to prevent the odds of accidental correlation due to evaluating multiple outcomes.

### 2.4. A Bioinformatics Analysis Revealed Promising MMP9 and APP/Aβ Partners for Further Scrutiny, Many of Which Are Already Linked with Retinopathy

**Figure 7:**
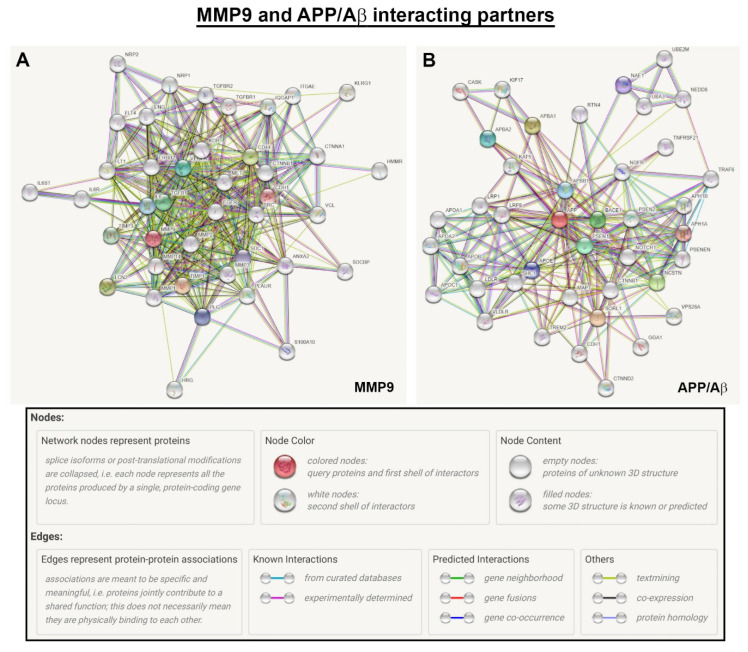
Bioinformatics tools (STRING database) were used to map MMP9- and APP/Aβ-interacting partners, which includes putative and experimentally demonstrable protein–protein interactions, containing molecules that jointly contribute to a shared function but without necessarily binding physically. (**A**) Notable MMP9 partners include several other members of the MMP family as well as TIMP3, all of which play important roles in retinopathies. Other partners include TGFBR1 and -2 as well as IL6 and its receptor subunits. VEGF-A and its receptor were also associated with MMP9. (**B**) Aβ partners were mapped to its parent precursor protein APP. Notable links were reported with β-secretase and a member of the TNF receptor family as well as an associated factor, which are all linked with retinal degeneration. We also found links with molecules involved in the clearance of Aβ. Other APP/Aβ partners that were identified in this manner includes a group of proteins that are associated with lipid metabolism, which is known to be dysregulated in the ageing retina and in retinopathies. Interestingly, cadherin-1 was associated with both MMP9 and APP/Aβ. Collectively, our bioinformatics analyses revealed candidate proteins, some of which could be assayed in the future to refine markers of ageing vs. AMD.

Although this study focused on elucidating the relationship of two potential biomarkers with age and AMD alongside lifestyle risk factors, both MMP9 and Aβ are also partners in a larger network of interactions with other proteins. We therefore used bioinformatics tools from the STRING database to identify protein partners of MMP9 (Figure 7A) and APP, the parent protein of Aβ (Figure 7B), with which they interact to varying extents, to identify other molecules that may contribute to AMD pathology. This analysis, which includes both putative and experimentally demonstrable protein–protein interactions, consists of molecules that jointly contribute to a shared function but without necessarily binding physically.

Mapping of MMP9 revealed potential targets such as cadherin-1 and related members, TIMP3, TGFBR1 and -2, as well as IL6 and its receptor subunits. VEGF-A and its receptor were also associated with MMP9. APP/Aβ partners included β-secretase and cadherin-1, as well as molecules associated with lipid metabolism. The aforementioned proteins represent promising targets for future scrutiny in studies of this kind.

## 3. Discussion

This exploratory study was carried out to determine the likely association of two disease-linked proteins, MMP9 and Aβ, with age, as well as with the presence and severity of AMD in a subset of patients. We also sought to ascertain the effects of potential confounding factors such as mean arterial pressure and the extent of smoking in the methodology of designing larger studies of this kind. The pursuit of identifying biomarkers of ageing and retinopathy presents considerable challenges, some of which include, but are not limited to: (1) deciding the appropriate number of participants, (2) selecting suitable target molecules and (3) identifying the best source, whether from serum, plasma or the type of ocular fluid (tears, aqueous or vitreous) from which measurements can be taken, as well as (4) the method of evaluation (in-house-developed vs. commercial ELISAs and multiplex assays). These considerations have to be offset by the number of participants that can realistically be recruited, the duration of the study and the technical constraints of measurements (for instance, some commercial ELISA-based methods are only optimised for measurements in certain types of samples such as cerebrospinal fluid). Another limitation includes instances where only partial information is available from patient records. An incomplete knowledge of the biology and the mechanisms of target molecules and their potential roles in complex diseases also pose challenges. Some studies collect information exclusively from non-invasive retinal scans, often involving large patient cohorts. Potential complications in setting-up such studies can be made even more difficult where ocular fluids and blood samples are also collected. However, the inclusion of biological material enables the direct measurements of chosen proteins to be determined at very high sensitivities, which adds considerable value, as we have demonstrated. Therefore, when powered sufficiently, studies of this kind can provide new insights into the biology of ageing as well as the diseased retina.

Here, we assessed the levels of MMP9 and Aβ in vitreous and serum/plasma samples from across the lifecourse of a control cohort alongside a subset of AMD patients. The functions of MMPs include the degradation of extracellular matrix proteins/glycoproteins, membrane receptors and cytokines as well as growth factors [10]. Of the 28 different MMPs in vertebrates, MMP9, alongside MMP2, belongs to a group containing three fibronectin-like inserts in the catalytic domain. MMP9 can act as a collagenases or a gelatinase and appears to be a biomarker for carotid atherosclerosis [11] and cancerous tumours, [12] along with being elevated in the plasma of Alzheimer’s patients [13]. Our previous work identified the rs42450006 variant upstream of MMP9 as being specifically associated with the NV but not the GA form of AMD [14]. Earlier work by another group demonstrated diminished levels of active MMP9 in the Bruch’s membrane of AMD patients leading to impaired matrix degradation [15]. Furthermore, hypoxic upregulation of MMP9 secretion, induced by pro-angiogenic vascular endothelial growth factor (VEGF) signalling, triggered the gene expression and secretion of VEGF in cultured RPE cells, indicative of a positive feedback mechanism between MMP9 and VEGF [16]. Our analysis of MMP9 in the vitreous and serum from controls and AMD patients showed no correlation with age or retinopathy. However, a major caveat is the limited number of individuals in the AMD cohort, as well as the small number of such patients from which measurements could be obtained. However, MMP9 levels were quantified in over 60% of the control cohort. To our knowledge, vitreous MMP9 levels across the lifecourse have not been reported before. Our data showed average values of 932.5 pg/mL and 813.7 pg/mL in the control and AMD groups, respectively. Serum MMP9 levels showed comparatively higher concentrations of 2228 pg/mL in control and 2386.8 pg/mL in AMD patients with no obvious correlations with aforementioned factors. An earlier study also showed no correlation of plasma MMP9 levels with increased age [17]. Serum MMP9 display a wide range of concentrations, with higher immature and active MMP9 levels alongside MMPs 1, 2, 7 and 8, compared to corresponding plasma samples [18], indicating potentially better sensitivities and outcomes from measurements in serum, which was also our preferred approach. A previous study that quantified MMP9 in the plasma of (a) controls, (b) patients with either large soft distinct drusen with pigmentary abnormalities, indistinct drusen or reticular drusen, or (c) NV AMD, reported values of 265 ng/mL, 659 ng/mL and 740 ng/mL, respectively, showing significantly elevated MMP9 in both early-intermediate AMD and NV groups vs. controls [19]. Another study that compared the plasma of control and AMD patients reported significantly higher MMP9 levels in GA patients (40 ng/mL vs. 76 ng/mL) [17]. The different outcomes of these studies compared to our findings may be due to several factors. For instance, both contained a lower number of control subjects compared to our study but had stratified AMD patients into two categories with each group having more patients. Differences may also arise as both studies used plasma whilst our work quantified MMP9 in serum. A further study reported increased levels of pro-MMP9 in plasma associated with NV AMD and with the risk allele of rs142450006 near MMP9 [20]. Of note, a study using serum from a Han Chinese cohort with large numbers for control, early and NV AMD groups, respectively, reported no association between MMP9 levels and retinopathy. However, increases in MMP2 and MMP9 were correlated with polypoidal choroidal vasculopathy, suggesting potentially different underlying mechanisms from AMD [21]. Additionally, immature/pro-MMP9 levels in tear samples were reported to be significantly elevated in conjunctivochalasis eyes relative to healthy controls [22]. However, the pre-analytical impact of blood collection requires careful consideration. For instance, the activity and concentration of MMP2 and TIMP2 showed no differences between plasma and serum, whilst levels and zymographic separation of MMP1, -8 and -9 as well as TIMP1 were strongly influenced by the presence of anticoagulants. Higher levels of TIMP1 and MMP1, -3 and -9 in serum compared to those in anticoagulant plasma suggested a release mechanism during coagulation and fibrinolysis. Hence, higher MMP levels in serum compared to plasma may not only relate to the disease status but also to the method of collecting blood samples [23]. Such considerations alongside the ability to quantify immature vs. active MMP9 levels to high sensitivities may provide better insights into its role across the lifecourse and in diseases such as AMD in the future.

The high concentrations of APP-derived fragments in ocular fluids also presented an opportunity to evaluate their relationships with age and retinopathy. The vitreous contains high Aβ levels relative to the aqueous [24], which prompted us to collect the former, although this also presented more difficulties in clinics. Cleavage of APP results in a mixture of Aβ polypeptides, predominantly Aβ_1–40_ and Aβ_1–42_, where different Aβ species show different solubility and biological properties. Aβ proteins are also found in various conformations including monomeric, oligomeric, prefibrillar and fibrillary forms, which adds to their diversity [25]. Histopathological studies of donor eye tissues show that Aβ deposits correlate with increased drusen loads and with AMD [4,26,27,28,29]. Given the important role played by Aβ in AD, potential findings from quantifying total Aβ in the vitreous could not only provide insights into any shared pathology in the retina–brain axis, but their quantification in blood could provide potential readouts in the systemic circulation. Although our results indicated that total Aβ in the vitreous had no correlation with age or AMD, another study reported high levels of Aβ_42_ (a component of total Aβ) in the aqueous to be correlated with NV AMD [30]. Interestingly, vitreous Aβ_1–42_ levels were diminished in glaucoma and diabetic retinopathy patients compared to control MH patients [31]. Lower levels of vitreous Aβ_1–40_ and Aβ_1–42_ were also reportedly correlated with poor cognitive function [32]. By contrast, elevated vitreous Aβ_1–40_ and Aβ_1–42_ were correlated with increased levels of neurofilament light chain in the vitreous [33]. The Aβ_42_:Aβ_40_ ratio of ~1:9 under normal physiological conditions in the brain is reported to shift to a higher percentage in favour of the latter in brains of familial AD patients. Interestingly, Aβ_1–40_ and Aβ_1–42_ directly interact and modify the behaviour of each other. Monomeric and fibrillar structures formed from Aβ_40_ and Aβ_42_ mixtures do not differ from those formed from either of these peptides alone. Instead, the co-assembly of Aβ_40_ and Aβ_42_ influences the aggregation kinetics by altering the pattern of oligomer formation [34]. The association of Aβ with worsening retinopathy and cognitive impairment is also reported in plasma and serum. For instance, serum APP and Aβ_1–40_ but not Aβ_1–42_ were correlated with GA [35]. Another study showed plasma Aβ_1–42_ and the Aβ_1–42_/Aβ_1–40_ ratio to be correlated with late stages of AMD [36]. Analysis of Aβ levels in serum vs. plasma by one-dimensional SDS-PAGE revealed insights into the most suitable method of pre-analytical sample collection. Quantification of samples either immediately or after 24 and 48 h at room temperature led to a significant loss of measurable peptide levels, which was most evident during the first 24 h of storage and more pronounced in serum compared to plasma. Hence, plasma may be more appropriate than serum for analysing Aβ [37], which was our chosen method for this study. Our findings show variable levels of total Aβ in the vitreous and serum of control patients that do not change significantly with age. Total Aβ concentrations in these fluids also do not appear to correlate with AMD status, although a major caveat is the relatively limited number of patients in our AMD subset. The study method may also have the unintended effect of influencing our data. For instance, AMD patients were generally older compared to control subjects, whilst all control subjects were patients at vitreoretinal clinics. To our knowledge, total Aβ levels across the lifecourse have not been reported before in the vitreous of non-AMD controls and in a subset of AMD patients. A previous study quantified soluble Aβ levels in the vitreous of 12 AMD patients vs. controls alongside levels of the receptor for advanced glycation end-products (RAGE), which serves as a receptor for Aβ. Soluble Aβ was detected in only 30% of AMD patients and 5% of controls. By contrast, the soluble RAGE receptor was detected in all samples but was significantly decreased in AMD patients [38]. Another study quantified Aβ_1–40_ and Aβ_1–42_ levels in the vitreous of healthy individuals (between the ages 55 to 101 years and within 12.5 ± 2 h of death), which reported values of 126.6 ± 57.3 pmol/g and 15.6 ± 5.7 pmol/g, respectively [39]. In our study, the total Aβ levels in plasma recorded amongst the control cohort was broadly in-line with values reported by others in healthy subjects [40,41]. Collectively, however, these results suggest that the quantification of certain Aβ species or indeed their ratios rather than total Aβ may be more accurately correlated with specific disease conditions.

As the odds of developing AMD are closely associated with modifiable risks such as the intake of unhealthy foods and alcohol as well as smoking [42,43,44,45,46,47], we used patient records to obtain lifestyle information in our cohorts including mean arterial pressure, smoking status and smoking pack years. Previous studies have examined the effects of blood pressure on the odds of developing AMD with inconsistent results [48,49], where hypertension plays an incompletely defined role in this multifactorial disease. This view is further supported by a recent report showing insufficient evidence to suggest a correlation between blood pressure and AMD [46], which was consistent with our findings. By contrast, smoking is a well-established modifiable AMD risk factor which has been consistently demonstrated in many epidemiological studies carried out in different populations. Smoking is associated with increased oxidative stress, a reduction in antioxidants and changes in choroidal endothelial cells linked with neovascularisation, as well as atherosclerosis or vasoconstriction, and promoting pro-inflammatory conditions in the retina [50]. It is, however, unclear how smoking affects the development of GA and NV phenotypes differently. A Mendelian randomisation approach was recently used to genetically predict smoking initiation and lifetime smoking with increased risks of developing advanced AMD. Similarly, cessation of smoking was linked with a decreased risk of advanced AMD. The study also revealed a greater potential association of smoking with NV AMD compared to GA [46]. Most studies have reported a dose–response effect between smoking and AMD. For instance, the Beaver Dam offspring study indicated that smoking 11 or more pack years was associated with early AMD [51], whilst the Rotterdam [52] and POLA [53] studies reported varying increased odds of developing advanced AMD in those who had smoked ≥10, ≥20, 20–39 or ≥40 pack years, with the risks of late AMD remaining high even 20 years after cessation of smoking. Our control and AMD cohorts contained a mixture of non-smokers and smokers with the latter group having smoked to varying extents. Although smoking was not correlated with vitreous or plasma Aβ levels, we observed a possible association with vitreous (*p* = 0.004) and serum (*p* = 0.005) MMP9 levels, though an increased threshold of *p* = 0.001 in our study meant that this was statistically insignificant.

An analysis of protein–protein interactions using bioinformatics tools revealed an extensive network of molecules linked with MMP9 and Aβ. Cadherin-1 was shown to interact with MMP9. A variant of CDHR1, another member of the cadherin superfamily that is specific to photoreceptors, was reportedly associated with inherited retinal dystrophies [54,55] and is a possible target for further scrutiny. Other interacting partners of MMP9 included fellow members of the MMP family such as MMP1, -2, -3 and -14, where links with retinopathies have already been discussed. A notable MMP9 partner is TIMP3, which we and others have shown to be linked with AMD as well as with Sorsby fundus dystrophy [14,56,57,58]. Serum TIMP3 levels in control subjects were reportedly diminished compared to AMD patients and in those that were negative for complement factor H duplication or deletion [59]. Furthermore, plasma TIMP3 levels in AD patients were significantly lower compared to those in healthy controls [60]. Other putative partners of MMP9 include TGFBR1, which we have shown to be associated with AMD [61] and TGFBR2. Interleukin (IL)-6 receptor subunits α and β alongside IL6 were also shown to be linked with MMP9. The role of IL6 in AMD pathogenesis is well established with a significant association of systemic IL6 levels with late AMD stages [62]. VEGF receptor-1 [63] and -2 [64] alongside VEGF-A were also associated with MMP9, all of which are important elements of retinal homeostasis. Neuropilins-1 and -2 are co-receptors for VEGF, which our bioinformatics analysis showed to be associated with MMP9. Neuropilin-1 was expressed in eight/nine surgically excised choroidal neovascular membranes [65] with a recent report showing reduced choroidal and retinal NV in mice lacking endothelial neuropilin-1 [66]. Vinculin, another target linked with MMP9, showed elevated levels in plasma of AMD patients [67].

*APP* encodes a cell surface receptor and transmembrane precursor protein, which is cleaved by secretases, resulting in several fragments that also includes a family of Aβ peptides associated with neurodegeneration [25]. This generates a plethora of different interactions with a multitude of molecules, the full extent of which can only be discerned by mapping against the parent APP protein. Our search revealed a network of such targets, of which several with specific relevance to Aβ and AMD have been highlighted. Presenilin-1 and -2 as well as the γ-secretase subunit PEN-2 are catalytic subunits of the γ-secretase complex, which generates Aβ. Although a PubMed search revealed no AMD studies specifically related to presenilin per se, knockout of β-secretase (*BACE1*), which also cleaves APP and was identified in our search, led to significant retinal pathology in a mouse model [68]. The triggering receptor expressed in myeloid cells 2 (TREM2), considered to be involved in the clearance of Aβ, was reported to be significantly diminished in AMD retinas [69]. Our search also identified a family of molecules associated with lipid metabolism, which includes the very low-density lipoprotein receptor (VLDR), the low-density lipoprotein receptor (LDLR), and apolipoproteins A-I and -II, C-I and E as well as B. Dysregulated lipid metabolism is known to play an important role in retinal pathology [70] including mediating effects of poor nutrition, which is a modifiable AMD risk factor. For instance, we have previously shown how an unhealthy “Western-style” high-fat diet alone causes salient early-intermediate AMD-like features in wildtype mice including changes to retinal lipids [47], and how AMD-linked disease pathways associated with an unhealthy diet can cause specific RPE dysfunction [71]. Dysregulated lipid metabolism also plays an important role in Alzheimer’s pathology [72]. Furthermore, our search identified the LDL-receptor-related protein-1 (LRP1) and -8 (LRP8), of which LRP1 is a major Aβ clearance transporter in the brain [73]. Other targets identified in this manner include members of the TNF receptor superfamily (TNFRSF21) and associated factor 6 (TRAF6) protein family, of which inhibition of the latter was shown to alleviate choroidal neovascularisation in a laser-induced mouse model [74]. Mapping APP partners identified cadherin-1, which, interestingly, our bioinformatics analysis also showed to be associated with MMP9.

## 4. Conclusions

In summary, we report that immature/active MMP9 and total Aβ concentrations across the lifecourse in control subjects and in a subset of AMD patients showed variable levels, which were unrelated to increasing age or retinopathy. Moreover, Aβ and MMP9 quantities in the vitreous and blood were not associated with mean arterial pressure, a modifiable factor whose role in retinal degeneration remains inconclusive. Assessment of vitreous and plasma Aβ with smoking, another modifiable factor but one that is strongly associated with disease, also showed no correlation. Interestingly, there was a hint that increased smoking pack years in control and AMD groups may have an association with immature and active forms of MMP9 in the vitreous (*p* = 0.004) and serum (*p* = 0.005), though this did not reach our elevated significance (*p* = 0.001) threshold. Close scrutiny of statistical parameters is therefore encouraged when evaluating reported associations. A PubMed search using the terms “age related macular degeneration”, “smoking” and “MMP9” yielded no results. We suggest recruiting larger cohorts to investigate these relationships further, as a caveat to this work is the low sample numbers, particularly amongst AMD patients. Both the control and AMD groups contained a mixture of ages, gender, ocular history, medication and lifestyle demographics, which could have the undesirable consequence of grouping heterogeneous phenotypes to mask potential effects. Furthermore, AMD patients were not genotyped, so may contain a mixture of risk genes. We recommend considering how certain lifestyle risks could influence biomarkers in different ways, and where possible grouping AMD subsets with distinct phenotypes and/or polymorphisms with which potential biomarkers could be mapped across the lifecourse. Obtaining additional information of modifiable factors such as diet from patient records, provided these are available, as well as categorisation by ethnicities and/or genotypes, could refine efforts. For instance, similar studies in non-Caucasian populations could yield different outcomes. We also recommend the exploitation of bioinformatics tools to identify partner proteins. Herein, we report links with molecules such as cadherin-1, MMPs, TGFBR1/2, IL6 and its receptor subunits, and VEGF-A and its receptor as well as β-secretase and those involved in lipid metabolism alongside others, which could be assayed to provide additional insights into common mechanisms and shared pathways.

## 5. Materials and Methods

### 5.1. Ethical Considerations for Recruiting Participants

The study was performed in accordance with the Research Governance Framework for Health and Social Care (2005) and the Declaration of Helsinki (2008) with approval by the local Research Ethics Committee (Rec Ref: 09/H0504/67) and Research and Development for University Hospital Southampton NHS Foundation Trust. In the majority of cases, participants received an information leaflet at least 24 h prior to surgery. However, if deemed appropriate, for instance in emergency cases, patients were recruited onto the study up to 1 h prior to surgery. Informed consent was obtained from all participants and the samples stored according to guidelines specified by the Human Tissue Act (HTA).

### 5.2. Study Cohort Eligibility and Clinical Examination

The study cohort was identified from vitreoretinal pre-operative lists of patients scheduled for vitrectomy, or vitrectomy following phacoemulsification and intraocular lens implantation at the University Hospital Southampton NHS Foundation Trust Eye Unit. The eligibility of patients was assessed via screening against the inclusion/exclusion criteria outlined in Table 1. Participants were recruited to the study only if the criteria were satisfied and were subject to informed consent. Funduscopy and OCT images from pre-operative assessments were examined independently by two ophthalmologists regarding AMD status. If the diagnoses were conflicting, the images were evaluated by a third consultant ophthalmologist. In total, 61 control subjects were recruited; however, 6 participants did not meet the study criteria (Table 1). The remaining 55 individuals did meet the inclusion criteria and were included in the study. We also recruited 12 AMD patients. The following information was obtained from patient records: age, gender and smoking status as well as smoking pack years (packs smoked per day x years smoked). Smoking was categorised according to published groupings as never smoked, light smoker and heavy smoker. Those who once smoked but are not current smokers were accordingly grouped as ex-light and ex-heavy smokers. Never smoked (0 SPY), ex-light smoker (0.01–7.5 SPY), ex-heavy smoker (≥ 7.5 SPY), light smoker (0.1–7.5 SPY) and heavy smoker (≥ 7.5 SPY). Additional information collected from patient records included the mean arterial pressure, the primary cause of surgery, the ocular history of both eyes, medical history and information on medication (Supplementary Table S1).

### 5.3. Sample Processing

Undiluted core vitreous biopsies (~0.4 mL) were obtained at the onset of pars planar vitrectomy. Prior to irrigation, the vitreous humour was manually aspirated from the centre of the vitreous cavity through a vitrectomy cutter into a 1 mL syringe with infusion in the off position. The volume of the biopsies was kept constant to minimise the potential for accidental inclusion of additional components from outside the vitreous core, which could otherwise compromise the proteome composition of the sample [75,76]. Upon receipt, samples were immediately placed on ice, distributed into 100 µL aliquots and stored at −80 °C. Serum and plasma samples were obtained via venous puncture into serum and EDTA tubes, respectively, and centrifuged at 1013× *g* for 10 min within 2 h of collection. The supernatants were collected and distributed into 500 µL aliquots and stored at −80 °C. Serum was collected for quantifying MMP9 in the blood, as previous studies found MMP9 levels to be higher in the serum than plasma, perhaps due to the delayed release from blood platelets and leucocytes during the clotting process [18]. In contrast, Aβ in blood was quantified in plasma, as Aβ is known to be more stable in serum compared to plasma in storage, including at room temperature for up to 48 h [37].

### 5.4. Enzyme-Linked Immunosorbent Assay for Quantifying MMP9

The levels of total MMP9 (immature and active MMP9 forms) in the vitreous humour and serum were quantified using a human solid-phase sandwich ELISA (ab100610, Abcam, Cambridge, UK) according to the manufacturer’s instructions. Prior to quantification, the samples were diluted 15-fold and 1000-fold for vitreous and serum, respectively. Briefly, 100 µL of the sample and known MMP9 standards were applied to wells of a microtitre plate that was pre-coated with the capture antibody and incubated overnight at 4 °C with gentle agitation. Following incubation, the plates were washed four times with wash solution, prior to applying 100 µL of biotinylated MMP9 detection antibody for 1 h at room temperature. Wells were washed an additional four times prior to the addition of 100 µL of HRP-streptavidin solution for 45 min at room temperature. The washing step was repeated and 100 µL of TMB one-step substrate reagent applied for 30 min in the dark at room temperature. Finally, 50 µL of stop solution was added and the optical density (O.D) measured at 450 nm using a microtitre plate reader (FLUOstar Optima, BMG LABTECH, Aylesbury, UK), which accounts for the 570 nm wavelength correction. Three technical replicates were measured for each sample. Data were analysed using MARS data analysis software (BMG LABTECH, Aylesbury, UK) and Microsoft Excel.

### 5.5. Enzyme-Linked Immunosorbent Assay for Quantifying Aβ

The level of total Aβ (Aβ_1–28_, Aβ_1–40_, Aβ_1–42_) in the vitreous humour and plasma was quantified using a Human Aβ_1-x_ solid-phase sandwich ELISA (Cat # 27729, IBL, Fujioka-Shi, Japan) according to the manufacturer’s instructions. Samples were diluted 9-fold prior to quantification. Briefly, 100 µL of the sample and known Aβ_1–40_ standards were applied to wells of a microtitre plate that was pre-coated with the capture antibody and incubated overnight at 4 °C. Following incubation, the plates were washed four times in wash buffer, before 100 µL of the labelled antibody solution was added for 1 h at 4 °C. Wells were washed an additional five times and 100 µL of the chromogen added for 30 min and the plates kept in the dark at room temperature. Finally, 100 µL of the stop solution was added and the O.D measured at 450 nm in a microtitre plate reader (FLUOstar Optima, BMG LABTECH, Aylesbury, UK), which accounts for a 570 nm wavelength correction. Three technical replicates were included for each sample. Data were analysed using MARS data analysis software (BMG LABTECH, Aylesbury, UK) and Microsoft Excel.

### 5.6. Bioinformatics Analysis

Protein partners (known and predicted) that could interact with MMP9 and the Aβ parent amyloid precursor protein (APP), as well as the likelihood of interaction, were mapped using bioinformatics tools from the STRING (Protein-Protein Interaction Networks Functional Enrichment Analysis) database (STRING Consortium 2021, Version 11.5 https://string-db.org/). ELIXIR Core Data Resources, Wellcome Genome Campus, Cambridgeshire, UK.

### 5.7. Statistical Analyses

The number of biological replicates for each analysis is shown in the accompanying figure legend. In some instances, fewer data points are shown, relative to the total number of patients recruited to the study in each group. This was due to incomplete patient information in medical records; for instance, where information could not be collated at clinical appointments due to time constraints or when consent was not granted by the patients. Similarly, data were excluded if ELISA measurements were below the standard curve. Statistical analyses were performed in SPSS Software (version 25, IBM Statistics) under the guidance of a medical statistician. For linear variables, the distribution of data was assessed for normality prior to correlation assessments with either Spearman’s rank or Pearson’s correlation coefficient. Correlation test data are expressed as Spearman’s rank correlation coefficient (rs) and *p* values. Kruskal–Wallis tests with a Hodges Lehmann estimator evaluated the association of linear variables with discrete data (2 groups or more) where data are presented as median (lower quartile, upper quartile). The association between linear variables of interest and AMD status was then assessed via linear regression analyses taking into account previously identified potentially confounding variables, where outcomes are reported as B coefficients and *p* values. Due to the assessment of multiple outcomes, the cut-off point for the level of statistical significance was reduced from *p* = 0.05 (Bonferroni) to *p* = 0.001 to minimise type 1 errors. Detailed descriptive statistics can be found in the Supplementary Table S2 and in the Supplementary Excel spreadsheet.

## Figures and Tables

**Table 1:** Inclusion and exclusion criteria used for identifying suitable control and AMD candidates. The study cohort was recruited from patients requiring vitreoretinal surgery. Hence, healthy control subjects included patients with previous or present underlying ocular conditions, which were assessed unlikely to affect the vitreous proteome. Furthermore, patients with systemic influences that could affect the vitreous proteome were also excluded from the control group. Patients with retinal vein occlusion and diabetic retinopathy were also excluded to prevent possible misdiagnosis with AMD. Abbreviations: AMD, age-related macular degeneration; CNV, choroidal neovascularisation; GA, geographic atrophy.

Inclusion Criteria	Exclusion Criteria
Age ≥ 50 Ethnicity: caucasian origin **Control group** Diagnosis confirmed by a consultant ophthalmologist for at least one of the following pathologies: • Cataract; • Epiretinal membrane; • Macular hole; • Vitreous floaters; • Vitreomacular traction syndrome. **Experimental group** Diagnosis confirmed by a consultant ophthalmologist for AMD: • Dry AMD; • Wet AMD. • Previous Wet AMD/Scar • AMD classification according to AREDS categories 2–4: Category 2: multiple small drusen/single intermediate drusen (63–124 µm) or RPE abnormalities. Category 3: extensive intermediate drusen, at least one large druse (≥125 µm) or GA not involving centre of fovea. Category 4: GA involving fovea and/or CNV.	Any ocular condition in which the vitreous proteome may be subject to systemic influence. Diagnosis confirmed by a consultant ophthalmologist for any of the following pathologies: • Diabetic retinopathy; • Retinal haemorrhage; • Retinal detachment; • Vitreous haemorrhage; • Retinal vein occlusion; • Wet AMD with vitreous haemorrhage. Age < 50 Participants lacking capacity to consent.

## Data Availability

All data have been included in the article and in the supplementary material. Reasonable requests for raw data will be considered by the authors before being made available to third parties.

## Supplementary Materials

The following supporting information can be downloaded at: https://www.mdpi.com/article/10.3390/ijms232314603/s1, Supplementary information includes anonymised patient information as well as individual MMP9 and Aβ measurements in participants (Supplementary Table S1) and the descriptive statistics (Supplementary Table S2 and Excel file).
