# Supplementary material for: An Exploratory Study Provides Insights into MMP9 and Aβ Levels in the Vitreous and Blood across Different Ages and in a Subset of AMD Patients
Source: Int J Mol Sci. 2022 Nov 23;23(23):14603. doi: 10.3390/ijms232314603 (PMC9736887; doi:10.3390/ijms232314603)
Supplement: Supplementary file 1 [file ijms-23-14603-s001.zip › Supplementary Material.pdf]

Supplementary Table S1

Number	Age	Gender	Smoking status	Smoking pack years	Mean arterial pressure	Primary cause of surgery	Previous ocular history	Medical history	Medication	Aβ vitreous (pg/ml)	Aβ blood (pg/ml)	MMP vitreous (pg/ml)	MMP blood (pg/ml)
**Control subjects**													
1	74	F	Never smoked	0	103.67	ERM, CAT		HYP	CV	1085.46		400.82	1563
2	72	F	Never smoked	0	98	ERM			CV, AP	581.47		399.91	1506.25
3	80	M	Never smoked	0	90.33	ERM		CRD, ART, HYP	CV, AP	960.47	265.6	262.62	1200.13
4	64	F	Ex heavy smoker	18.75	88	VMT			MET	1830.34	44.18	289.55	958.49
5	58	F	Ex heavy smoker	7.5	91.67	ERM				464.3	19.32	1752.75	1217.83
6	76	F	Ex light smoker	0.5	91.67	ERM		ART, DBT	CV, MET, AP, COAG, MUSC	1346.19	574.43	630.58	1290.88
7	71	F	Never smoked	0		VF	CAT, GLA		CV, MET, AP		320.72		1947.3
8	60	F	Never smoked	0		VF	ERM, CAT, VMT		MET, NRV, HRMNS	1144.07	170.79	437.3	1780.69
9	64	F	Ex heavy smoker	18	79.33	ERM		ART, DEP	CV, MET, NRV	971.46	167.91	214.07	1899.5
10	72	M	Heavy smoker	15		ERM		ART		2342.9	833.67	191.77	1594.28
11	74	F	Never smoked	0		ERM		HYP	CV	619.02	178.77	263.11	899.65
12	78	F	Never smoked	0	85.67	CAT, VMT		CRC, ART, HYP	CV, MET, AP, COAG	897.07	410.27	295.58	487.81
13	79	M	Never smoked	0	83.33			ART	CV, MET, NRV, AP, COAG, GUSSH	2640.17	1226.49	703.04	1214.88
14	63	F	Ex heavy smoker	7.5	75.33	ERM		ART	NRV	1698.33	306.35	184.59	1758.51
15	74	F	Never smoked	0	93.67	MH	CAT	CRC		927.85		123.4	
16	72	M	Heavy smoker	10	74	ERM				1256.12		328.05	
17	76	F	Never smoked	0	84.67	ERM	CAT	ART	MUSC	476.92	316.96	192.8	1556.78
18	78	M	Heavy smoker	15	100	MH		CRD, HYP	CV, MET, AP, GUSSH	1417.93		697.05	
19	79	F	Ex heavy smoker	20	118	VMT			CV, MET, NRV, COAG	2048.9		2208.59	
20	77	F	Never smoked	0	104	ERM			CV, MET, NRV, AP	1319.06	290.92	4844.79	3076.08
21	63	F	Never smoked	0	125	ERM		ANX	CV	734.29	519.8	400.58	1732.14
22	76	M	Ex light smoker	1	99.33	ERM			CV	600.28	218.62	1854.78	1428.04
23	66	F	Light smoker	1.6	98	MH			MET	2833.81	1625.68	0.45	2354.17
24	82	F	Light smoker	6	102.67	ERM		CRC	CV	21.63	3102.18		1230.07
25	75	F	Ex light smoker	6	102	ERM		ART	MET, AP, COAG, IMU	3030.97		600.44	
26	77	F	Not declared	0	105.67	ERM		ANX	NRV	2808.91	453.07	567.39	2089.63
27	78	F	Ex heavy smoker	22.5	104	MH		ART, HYP	CV, AP, COAG, HRMNS	239.25	368.82		2301.25
28	65	F	Ex light smoker	0		MH		ANX		971.28	1774.54		1971.97
29	70	F	Heavy smoker	27.5	91	ERM		ART, ANX	MET, NRV	257.06	296.69		2370.07
30	58	F	Heavy smoker	11.4	100	MH				80	798.45		4003.27
31	78	M	Never smoked	0	106	ERM	CAT		CV	12	136.77		2576.01
32	72	F	Never smoked	0	97.33		MH	HEP, ART, HYP, ANX	NRV, AP	814.01	249.61	827.63	4033.01
33	53	M	Never smoked	0	78	VF				109.37	1375.2	122.89	4794.21
34	72	M	Never smoked	0	92	ERM, CAT, MH		HYP	CV, COAG	586.5	1780.81		2642.98
35	76	F	Never smoked	0	93.67		CAT	CRC, ASM	AP, RSP	2103.18	149.97	895.91	1842.25
36	67	F	Never smoked	0		ERM		ART, ASM	AP, RSP	1583.69	148.53	97.72	1466.49
37	82	F	Ex light smoker	2.5		ERM, CAT		CRD, HYP	CV, COAG, GUSSH	41.69	273.02		1652.63
38	71	F	Ex heavy smoker	75	78.33	MH			CV, MET, NRV, COAG	1917.43	343.66		4430.5
39	70	M	Ex light smoker	0.75	109.67	MH			CV	48.65	267.28		620.92
40	82	M	Ex light smoker	2.75	90	ERM	CAT	ART	NRV, COAG, GUSSH, IMU	1603.18	180.54	160.54	1331.35
41	66	F	Never smoked	0	95.67	MH	CAT			918.29	357.58	2330.96	3329.66

42	71	M	Ex light smoker	0.05	110.67				CV, COAG	1254.62	175.44		1288.64
43	79	F	Never smoked	0	99.33	MH			MET	106.12	233.87		2450.5
44	64	F	Heavy smoker	30						978.79	277		5831.68
45	74	F	Never smoked	0		ERM		CRD, ART	CV, MET, AP, MUSC	424.72	310.62	362.43	5367.81
46	73	M	Ex light smoker		94.66	ERM, CAT			GUSSH		839.62	590.37	3153.24
47	66	M	Ex heavy smoker	37.5		CAT, VMT	ERM, CAT	CRC, CRD, ART, HYP, DBT	CV, COAG, GUSSH	816.08	208.58	6803	3910.97
48	70	F	Never smoked	0	105.66	ERM, CAT	ERM, CAT		CV	1395.25	200.86	1669.6	1647.29
49	52	M	Ex light smoker	3		CAT	CAT	CRD	CV, AP	193.58	3608.1		
50	90	F	Ex light smoker	20		ERM, CAT, VMT	CAT, VMT	ART, HYP, DEP	CV, NRV, MUSC	1579.41	271.83		
51	64	F	Ex light smoker	2.5		ERM, CAT, MH, VMT	CAT, MH	DEP, ANX	MET, NRV, MUSC	1536.92	235.07		
52	75	M	Ex light smoker	25	87.33	MH, VMT	CAT, MH, PVD	HYP	CV, MET, AP	3086.91	305.53		
53	57	M	Ex light smoker	1.6	98.66	ERM				769.42	263.66		
54	61	F	Never smoked	0	94.66	ERM			MET, SENS	2255.25	440.33		
55	86	F	Ex light smoker	3	99	ERM		CRD, ART, HYP	CV, MET, COAG, SENS	2457.75	650.7		

AMD patients

Number	Age	Gender	AMD type	Smoking status	Smoking pack years	Mean arterial pressure	Primary cause of surgery	Previous ocular history	Medical history	Medication	Aβ vitreous (pg/ml)	Aβ blood (pg/ml)	MMP vitreous (pg/ml)	MMP blood (pg/ml)
1	81	F	Dry AMD	Never smoked	0	127.33	ERM			CV, MET, AP, COAG	803.26	316.92		1468.93
2	78	M	Dry AMD	Ex light smoker	1.5	93	MH	ERM	HYP	CV, AP	1984.87		469	
3	78	M	Early AMD	Never smoked	0	86	ERM	PVD		CV	786.32	351.08	717.59	3264.19
4	83	M	Early AMD	Never smoked	0	107.33				CV, COAG	1758.62	314.37	1224.9	1770.24
5	72	F	Early AMD	Ex heavy smoker	18	98	ERM			CV	773.87		843.35	
6	71	M	Dry AMD	Ex heavy smoker	18.75	89.33	ERM	CAT, PVD	CRD, ART, HYP	NRV, COAG, IMU		1732.15		3043.69
7	84	F	Wet AMD	Ex heavy smoker	10	106.67	RD	CAT	HYP	CV, NRV, COAG	3240.12	339.41		
8	93	F	Dry AMD	Ex light smoker	0.01	85.33	VMT	CAT		MET, RSP		472.75		
9	90	F	Early AMD	Never smoked	0	90.33	ERM	CAT	CRD	CV, MET, AP, GUSSH	26.08	646.08		
10	72	F	Dry AMD	Never smoked	0		CAT	CAT	ART, DEP	NRV	48.58	550.25		
11	68	M	Dry AMD	Never smoked	0		ERM, CAT	ERM, CAT	LNG		2470.39	156.92		
12	81	F	Dry AMD	Ex light smoker	5	102.66	ERM, CAT, MH, VMT	ERM, CAT, MH	CRD, ART, HYP	CV, MET, MUSC	863.58	541.92		

Primary Cause of surgery and Ocular History

Condition	Key
Epiretinal membrane	ERM
Cataract	CAT
Macular Hole	MH
Retinal Detachment	RD
Vitreous Floaters	VF
Vitreomacular Traction	VMT
Vitreous Detachment	VD
Glaucoma	GLA
Posterior Vitreous detachment	PVD

Medical history

Condition	Key
Hypertension	HYP
Cardiopathies	CRD
Arthritis	ART
Depression	DEP
Diabetes	DBT
CRC	CRC
Hepatic	HEP
Asthma	ASM
Lung	LNG

Medication

Target	Key
Cardiovascular	CV
Hypertension	HYP
Metabolic	MET
Coagulation modifier	COAG
musculoskeletal	MUSC
nervous	NRV
Genito, Urinary system and Sex Hormones	GUS
Hormones, excl. Sex Hormones	HRMNS
Sensory	SENS
Immunosuppressants	IMU
Respiratory	RSP

Supplementary Table S2

DESCRIPTIVE STATISTICS

	25%	Median	75%	N	SE	SD
Abeta Vitreous	935.0867	971.3675	1397.2155	54	115.20124	846.55273
Abeta Plasma	363.43480	301.10900	785.2496	48	104.838	726.34093
MMP9 Vitreous	37.5902	502.3435	11609.7005	38	2855.6296	17603.283
MMP9 Serum	1838.8702	1780.6853	2617.0700	43	192.8075	1264.3233
MAP	92.4405	97.333	99.0776	43	1.64441	97.333
Smoking pack/year	3.8722	0.7500	11.0333	55	1.78592	13.24477
Age	65.5000	72.0000	77.0000	56	1.075	8.041
MMP9 Vitreous <40000	454.4675	400.815	1405.4382	35	223.97055	1384.19

PRD013 Excluded, AMD Excluded, Outliers in MMP9 Vitreous Excluded for <40000.

Abeta Vitreous

	Valid	FALSE	% false	25%	Median	75%	SE	SD	TRUE	% true	25%
Female									38		581.46
Male									16		390.04
Never smoked									22		581.465
Ex Light Smoker									14		600.28
Ex Heavy Smoker									9		773.87
Light Smoker									2		21.6
Heavy Smoker									6		257.06
pcsERM	49	18	36.73469388	193.58	923.0652	1830.335	221.66025	940.42479	31	63.26531	583.982
pcsCAT	49	39	79.59183673	470.6125	960.465	1650.7573	137.22721	856.98363	10	20.40816	586.
pcsMH	49	36	73.46938776	529.1942	965.96	1593.436	133.82507	802.95041	13	26.53061	239.253
pcsRD	48	48	100	470.6125	944.155	1593.436	123.68852	856.9392	0	0	
pcsVF	49	47	95.91836735	529.1942	960.465	1593.436	124.72182	855.04972	2	4.081633	109.370
pcsVMT	49	42	85.71428571	424.72	923.0652	1417.93	128.89069	835.30714	7	14.28571	1216.994
pcsPE	49	49	100	476.9233	960.465	1583.6907	121.43987	850.07906	0	0	

[illegible]

PRESENTING DATAS

[illegible]

STATISTICS

[illegible]
